# *AQP1* and *SLC4A10* as candidate genes for primary open-angle glaucoma

**Published:** 2010-01-20

**Authors:** Wenjing Liu, Yutao Liu, Xue-Jun Qin, Silke Schmidt, Michael A. Hauser, R. Rand Allingham

**Affiliations:** 1 Center for Human Genetics, Duke University Eye Center, Duke University Medical Center, Durham, NC; 2 Department of Ophthalmology, Duke University Eye Center, Duke University Medical Center, Durham, NC

## Abstract

**Purpose:**

Recent evidence supports the role of reduced cerebrospinal fluid (CSF) pressure in the pathogenesis of primary open-angle glaucoma (POAG). We investigated the association of variants in two candidate genes that are important in CSF production, aquaporin 1 (*AQP1*) and solute carrier family 4, sodium bicarbonate transporter, member 10 (*SLC4A10*), with POAG in the Caucasian population.

**Methods:**

POAG subjects (n=382) met the criteria of glaucomatous optic neuropathy with consistent visual field loss. Intraocular pressure was not used as an inclusion criterion. Control subjects (n=363) did not meet any of the inclusion criteria and had no family history of glaucoma. Eleven tagging single nucleotide polymorphisms (SNPs) for *AQP1* and *SLC4A10* were genotyped in the POAG and control subjects, using allelic discrimination assays. Genotype frequencies were compared between the POAG and control subjects, using logistic regression adjusted for gender.

**Results:**

There was no statistically significant difference in genotype frequencies between POAG and control subjects for any of the tested SNPs in *AQP1* and *SLC4A10* (p>0.05).

**Conclusions:**

There was no association between common sequence variants in the *AQP1* or *SLC4A10* genes and POAG in the Caucasian population. This is the first study to investigate the association between these two candidate genes and increased risk for POAG.

## Introduction

Primary open-angle glaucoma (POAG; OMIM 137760) is the most common form of glaucoma, which is the leading cause of irreversible blindness worldwide [1,2]. POAG is characterized by a chronic optic neuropathy and progressive loss of retinal ganglion cells, leading to specific visual field defects in the absence of a known secondary cause [3,4]. Several risk factors have been associated with POAG, including elevated intraocular pressure (IOP), advanced age, black race, and family history [5]. To date, four causative genes for POAG from 11 candidate chromosomal loci have been identified, including *MYOC* (myocilin), *OPTN* (optineurin), *WDR36* (WD repeat domain 36), and *CYP1B1* (cytochrome P450, family 1, subfamily B, polypeptide 1), but these four genes together account for less than 10% of POAG cases [6-10].

Recent studies have provided strong support for the theory that reduced cerebrospinal fluid (CSF) pressure may play a role in the pathogenesis of POAG. In a retrospective review of patients with a history of lumbar puncture at the Mayo Clinic (Rochester, Minnesota), Berdahl et al. [11] reported that the mean CSF pressure was significantly lower in POAG patients when compared with nonglaucomatous control patients. A prospective study conducted by Ren et al. [12] confirmed that the mean CSF pressure is lower in POAG patients and in normal tension glaucoma (NTG) patients when compared with nonglaucomatous control patients. A decreased CSF pressure can lead to an increased translaminar pressure difference, which is defined as the pressure difference between IOP and CSF pressure [13]. When the translaminar pressure difference is abnormally increased, axoplasmic flow is disrupted, leading to retinal ganglion cell death and optic disc changes characteristic of glaucoma [14-18]. These findings lend support to the hypothesis that a decreased CSF pressure resulting in an increased translaminar pressure difference may contribute to the pathogenesis of glaucoma.

The candidate gene approach is used to study genes that are hypothesized to play a role in the etiology of a complex human disease with genetic contributions. This approach has been successful in identifying genes, such as complement factor H in age-related macular degeneration [19]. In this study we investigated aquaporin 1 (*AQP1*) and solute carrier family 4, sodium bicarbonate transporter, member 10 (*SLC4A10*) as candidate genes for POAG. *AQP1* maps to chromosomal location 7p14 and *SLC4A10* maps to chromosomal location 2q23-q24, neither of which is at a known chromosomal locus for POAG listed by the Human Genome Organization [20-23]. Both of these genes are expressed in the choroid plexus and have been shown to be important in CSF production [20-29]. Knockout mouse models of these genes demonstrate a significant reduction in CSF production and intracranial pressure [22,28].

We hypothesized that *AQP1* and *SLC4A10* have a role in the pathogenesis of POAG because of their function in CSF production and their effect on the translaminar pressure difference. In this study, we investigated the association between sequence variants of these genes with increased risk for POAG in the Caucasian population.

## Methods

### Subjects

This study was reviewed and approved by the Institutional Review Board of Duke University Medical Center (Durham, NC) and adhered to the tenets of the Declaration of Helsinki. Informed consent was obtained from all study participants. Study subjects were recruited from the Duke University Eye Center (Durham, NC) for a total of 382 subjects with POAG and 363 control subjects. Subjects with POAG were unrelated and met the following inclusion criteria: 1) age of onset greater than 30 years; 2) glaucomatous optic neuropathy in both eyes; and 3) visual field loss consistent with optic nerve damage in at least one eye [30]. Glaucomatous optic neuropathy was defined as a cup-to-disc ratio greater than 0.7 or focal loss of the nerve fiber layer resulting in a notch, associated with a glaucomatous visual field defect. Visual fields were performed using standard automated perimetry or frequency doubling test [2]. IOP was not used as an inclusion criterion. The exclusion criteria for POAG subjects included the diagnosis of a secondary form of glaucoma or a history of ocular trauma. The control subjects were examined by the same glaucoma subspecialist (RRA) who examined the POAG subjects. The control subjects were unrelated and met the following criteria: 1) no first-degree relative with glaucoma; 2) IOP less than 21 mmHg in both eyes without treatment; 3) no evidence of glaucomatous optic neuropathy in either eye; and 4) normal visual field in both eyes.

### Genomic DNA genotyping

Genomic DNA was extracted from peripheral blood samples via alcohol and salt precipitation using Gentra Systems PUREGENE DNA Purification Kit (Qiagen, Valencia, CA). Based on the genotype data from the HapMap Project, HaploView software version 4.1 (Broad Institute, Boston, MA) was used to select 11 tagging single nucleotide polymorphisms (SNPs) for *AQP1* and *SLC4A10* in the Caucasian population, using an r^2^ threshold of 0.6 and a minor allele frequency threshold of 0.05 [31]. TaqMan allelic discrimination assays were used for genotyping with Assays-On-Demand and Assays-By-Design products, according to the standard protocols from the manufacturer (Applied Biosystems, Foster City, CA) [32]. For quality control purposes, two Centre d'Etude du Polymorphisme Humain standards (CEPH, Paris, France) and quality control samples were placed within and across 384-well plates (Applied Biosystems, Foster City, CA), and laboratory personnel were blinded to the location of these samples. Genotype submission to the analysis database required matching genotypes for all quality control samples and at least 95% genotyping efficiency.

### Statistical analysis

Analysis of Hardy–Weinberg equilibrium was performed separately for POAG and control subjects with Genetic Data Analysis (GDA) software [33]. Pairwise linkage disequilibrium (LD) between SNPs was calculated with the GOLD software [34]. Genotype frequencies in POAG and control subjects were compared by logistic regression with adjustment for gender using SAS software (SAS Institute Inc., Cary, NC). SNP genotypes were coded according to a log-additive risk model, which assumes that the risk from carrying a single copy of the variant (minor) allele is midway between that of zero copies (reference genotype) and of two copies on the logarithmic scale. Power calculations were performed using QUANTO software according to previously described methods, assuming a population prevalence of 10% and a log-additive risk model [35].

## Results

A total of 382 POAG subjects and 363 control subjects were included in the Caucasian data set. Of the POAG subjects, 15 subjects had NTG, defined as a maximum IOP <22 mmHg. The mean age of onset of POAG in the experimental subjects was 57.6±14.2 years, and the mean age of control subjects at the time of ophthalmologic exam was 64.8±9.3 years. The experimental group was 49.7% female, and the control group was 59.8% female.

**Figure 1:**
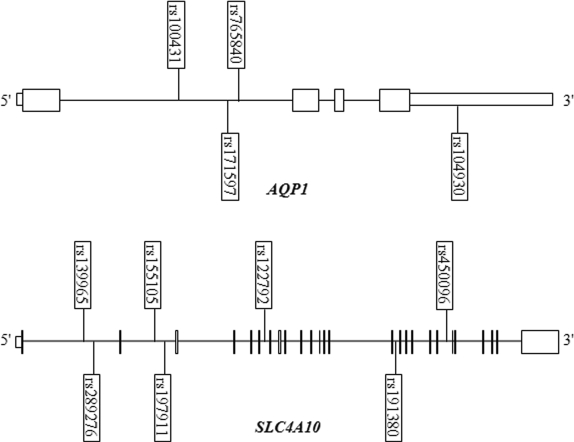
Schematic of aquaporin-1 (*AQP1*) and solute carrier family 4, sodium bicarbonate transporter, member 10 (*SLC4A10*) genes. The locations of the genotyped tagging single nucleotide polymorphisms (SNPs) identified using HaploView software in the *AQP1* and *SLC4A*10 genes are shown in relation to the exons that are depicted as vertical boxes or lines and the introns that are depicted as a horizontal line.

A total of 11 tagging SNPs were selected to cover the LD blocks of *AQP1* and *SLC4A10*, using HaploView software (Figure 1). All of these were in Hardy–Weinberg equilibrium (p>0.01) in the Caucasian controls. We did not observe statistically significant pair-wise LD (with an r^2^ cut-off of 0.6) between the four tagging SNPs of *AQP1* or the seven tagging SNPs of *SLC4A10*. No significant genotype frequency differences between cases and controls were detected in either *AQP1* or *SLC4A10* (Table 1).

**Table 1:** Minor allele frequencies of tagging SNPs in the *AQP1* and *SLC4A10* genes in Caucasian POAG and control subjects

**Candidate gene**	**SNP**	**Allele**	**Controls (n=363)**	**POAG** **(n=382)**	p-value*
*AQP1*	rs1004317	G	0.404	0.399	0.786
	rs17159702	G	0.292	0.265	0.297
	rs765840	A	0.067	0.077	0.468
	rs1049305	C	0.412	0.367	0.078
*SLC4A10*	rs1399650	C	0.157	0.152	0.739
	rs2892769	C	0.451	0.439	0.639
	rs1551051	T	0.267	0.237	0.146
	rs1979112	G	0.121	0.141	0.221
	rs1227929	T	0.409	0.411	0.901
	rs1913807	T	0.207	0.213	0.574
	rs4500960	A	0.490	0.483	0.726

The asterisk indicates that the p-value is from logistic regression with adjustment for sex, using log-additive coding of SNP genotypes; SNPs: single nucleotide polymorphisms; POAG: primary open-angle glaucoma; *AQP1*: aquaporin-1; *SLC4A10*: solute carrier family 4, sodium bicarbonate transporter, member 10.

For the three SNPs with lower minor allele frequencies (ranging from 7% to 16% for rs765840, rs1399650, and rs1979112), our Caucasian data set had >94% power at a two-tailed significance level of 5% to detect an odds ratio of 2 or greater. For all other SNPs, our Caucasian data set had >90% power at a two-tailed significance level of 5% to detect an odds ratio of 1.5 or greater.

## Discussion

Recent studies have shown that CSF pressure is reduced in POAG subjects as compared to control subjects, suggesting that CSF pressure may play a role in the pathogenesis of POAG [11,12]. Decreased CSF pressure leads to an increase in the translaminar pressure difference. Elevations of IOP also increase translaminar pressure differences, which have been shown to disrupt axoplasmic flow and cause retinal ganglion cell death in a glaucomatous pattern [14-18]. Two genes that are expressed in the choroid plexus and have been shown to be important in CSF production are *AQP1* and *SLC4A10* [20-29]. These two genes were selected out of many other genes implicated in CSF production because of their selective expression in the choroid plexus, their functional significance in water and ion transport, and their demonstrated role in CSF production in knockout mouse models [20-29]. However, we did not detect an association between common tagging SNPs of *AQP1* or *SLC4A10* and POAG in this study.

In this study we focused on the potential role of genes associated with CSF production in glaucoma. It is important to note that *AQP1* is also expressed in the trabecular meshwork and Schlemm’s canal cells located within the conventional aqueous outflow tract of the eye [36], while *SLC4A10* is not [37]. *AQP1* is believed to improve cell viability in the setting of mechanical strain, but the degree to which *AQP1* contributes to bulk outflow in the conventional outflow tract of the eye is uncertain [38-40].

It is known that *AQP1* and *SLC4A10* are major contributors to CSF production. Due to the functional importance of *AQP1* and *SLC4A10* in CSF production, sequence variants within these two genes might be expected to be more prevalent in NTG patients since both a retrospective and a prospective study showed a trend of NTG patients having lower CSF pressure than POAG subjects [12,41]. Our Caucasian data set is composed primarily of POAG subjects with high tension glaucoma, and only 15 subjects had NTG. Therefore, this study did not have sufficient power to detect an association between *AQP1* or *SLC4A10* and an increased risk of NTG.

Our large sample size provided adequate statistical power to detect a moderate or strong association between common sequence variants of these two genes and an increased risk of POAG in the Caucasian population. However, we cannot rule out the presence of such an association in populations of different ancestry. It is also possible that only rare sequence variants in these genes, which our study was not designed to detect, have an appreciable effect on CSF production and pressure. Additionally, mutations identified in different regions of *AQP1* have been shown to reduce water and ion transport function [42-43]. Targeted mutations, such as an exon deletion in *SLC4A10* knockout mice, resulted in an 88% reduction in brain ventricle size from decreased CSF production as compared to wild-type mice [28]. The effect of knockout models of these genes on the optic nerve has not been described to date. Further studies would be necessary to identify naturally occurring mutations in *AQP1* and *SLC4A10* that lead to decreased CSF pressure.

In conclusion, we did not find an association between common sequence variants in *AQP1* and *SLC4A10* and high tension POAG in our Caucasian data set. Further studies are needed to determine if variants within these two genes are associated with NTG only or if rare sequence variants with a greater effect on CSF pressure may play a role in POAG in populations of Caucasian or non-Caucasian origin.
